# Burning in Banksia Woodlands: How Does the Fire-Free Period Influence Reptile Communities?

**DOI:** 10.1371/journal.pone.0034448

**Published:** 2012-04-05

**Authors:** Leonie E. Valentine, Alice Reaveley, Brent Johnson, Rebecca Fisher, Barbara A. Wilson

**Affiliations:** 1 School of Veterinary and Biomedical Sciences, Western Australian Centre of Excellence for Climate Change, Woodland and Forest Health, Murdoch University, Perth, Western Australia, Australia; 2 Gnangara Sustainability Strategy, Department of Environment and Conservation, Perth, Western Australia, Australia; 3 Oceans Institute, University of Western Australia, Perth, Western Australia, Australia; Australian Wildlife Conservancy, Australia

## Abstract

Fire is an important management tool for both hazard reduction burning and maintenance of biodiversity. The impact of time since last fire on fauna is an important factor to understand as land managers often aim for prescribed burning regimes with specific fire-free intervals. However, our current understanding of the impact of time since last fire on fauna is largely unknown and likely dependent on vegetation type. We examined the responses of reptiles to fire age in banksia woodlands, and the interspersed melaleuca damplands among them, north of Perth, Western Australia, where the current prescribed burning regime is targeting a fire-free period of 8–12 years. The response of reptiles to fire was dependent on vegetation type. Reptiles were generally more abundant (e.g. *Lerista elegans* and *Ctenophorus adelaidensis*) and specious in banksia sites. Several species (e.g. *Menetia greyii*, *Cryptoblepharus buchananii*) preferred long unburnt melaleuca sites (>16 years since last fire, YSLF) compared to recently burnt sites (<12 YSLF). Several of the small elapids (e.g. the WA priority listed species *Neelaps calonotus*) were only detected in older-aged banksia sites (>16 YSLF). The terrestrial dragon *C. adelaidensis* and the skink *Morethia obscura* displayed a strong response to fire in banksia woodlands only. Highest abundances of the dragon were detected in the recently burnt (<7 YSLF) and long unburnt (>35 YSLF) banksia woodlands, while the skink was more abundant in older sites. Habitats from a range of fire ages are required to support the reptiles we detected, especially the longer unburnt (>16 YSLF) melaleuca habitat. Current burning prescriptions are reducing the availability of these older habitats.

## Introduction

Fire is a management tool for landscape custodians of conservation reserves and remnant vegetation [1], [2]. Prescribed burning is used to reduce the threat of wildfires [3], [4], [5], especially on the outskirts of towns and cities adjoining remnant vegetation where expanding infrastructure to accommodate a growing human population is associated with a high risk in wildfires, as evident in Mediterranean-type ecosystems [6], [7]. As the role of fire in determining environmental and biological heterogeneity is well established [4], [8], [9], [10], conservation managers also employ prescribed burning for maintenance of biodiversity. Research on the impacts of fire on flora [11], [12], [13], [14] and fauna [15], [16], [17] has been substantial. However, our understanding of the responses of fauna to different components of prescribed burning is still minimal [18], and often difficult for land managers to adapt to burning prescriptions.

All components of fire regimes (e.g. seasonality, intensity and frequency) influence the impacts of fire on fauna. However, an important consideration arising from fire and fauna research is that the fire-free period may be instrumental in structuring faunal communities [17], . Long-term studies of reptile assemblages in arid regions [17],[21],[22], forests [23], [24], and sand-pine scrub [25] consistently indicate a reptile succession with time since last fire as different species dominate when appropriate habitat presides. The habitat accommodation model of succession [19] indicates that as vegetation structure recovers from a fire, there will be a corresponding predictable sequence of faunal recovery. As vegetation structure affects habitat and resource availability for fauna, time since last fire may affect resources such as refuges, food availability, predator susceptibility and thermal buffering. For example, burning within a short successive time frame in tropical savannas reduces the availability of an important fruiting shrub upon which frugivorous birds feed [26].

Our study was undertaken on a Mediterranean-type ecosystem located on the Swan Coastal Plain in the south-west Western Australia (SWWA). The SWWA region is internationally recognised because of its high levels of biodiversity and endemicity, and the high degree of threatening processes, such as habitat loss associated with urban and rural development [27]. Ecosystems on the Swan Coastal Plain are considered some of the most flammable in SWWA, due to the lengthy period of the year that the vegetation is combustible, and plant growth adaptations that result in rapid accumulation of vegetation after fire [28]. Conservation estate within the region contains banksia woodland interspersed with melaleuca damplands with high biodiversity values, particularly of the ground-dwelling vertebrates [29], [30]. In order to reduce the risk of wildfires [31], [32], the prescribed burning regime for the area is targeting a fire-free period of 8–12 years. However, inappropriate fire regimes have been recognised as a major threatening process on the Swan Coastal Plain [33], [34], [35]. Studies in urban remnants of vegetation in the Perth region have shown that lizard diversity was greatest in areas that have remained unburnt the longest [36], indicating a susceptibility of some reptile species to frequent burning.

In our study, we examine the responses of reptiles, using reptile species richness, abundance and community structure as variables, to time since last fire in two habitat types, banksia woodlands and melaleuca damplands on the Swan Coastal Plain. The key questions we addressed were: 1) Do reptile communities vary between vegetation types and fire-age categories based on prescribed burning management targets?; 2) Are there detectable seral responses of reptiles to time since last fire and microhabitat variables, such as litter cover; and 3) What are the implications of these relationships for current fire management objectives. Understanding the responses of reptiles to time since last fire in large remnant vegetation patches may provide important information for fire management that is currently in practice in this region.

## Methods

### Ethics statement

Data collected adhered to the legal requirements of Australia (WA Department of Environment and Conservation Scientific Purposes Permit: SC 000826) and to the ethical guidelines for treatment of animals by the WA Department of Environment and Conservation (AEC 37/2007).

### Study area

**Figure 1:**
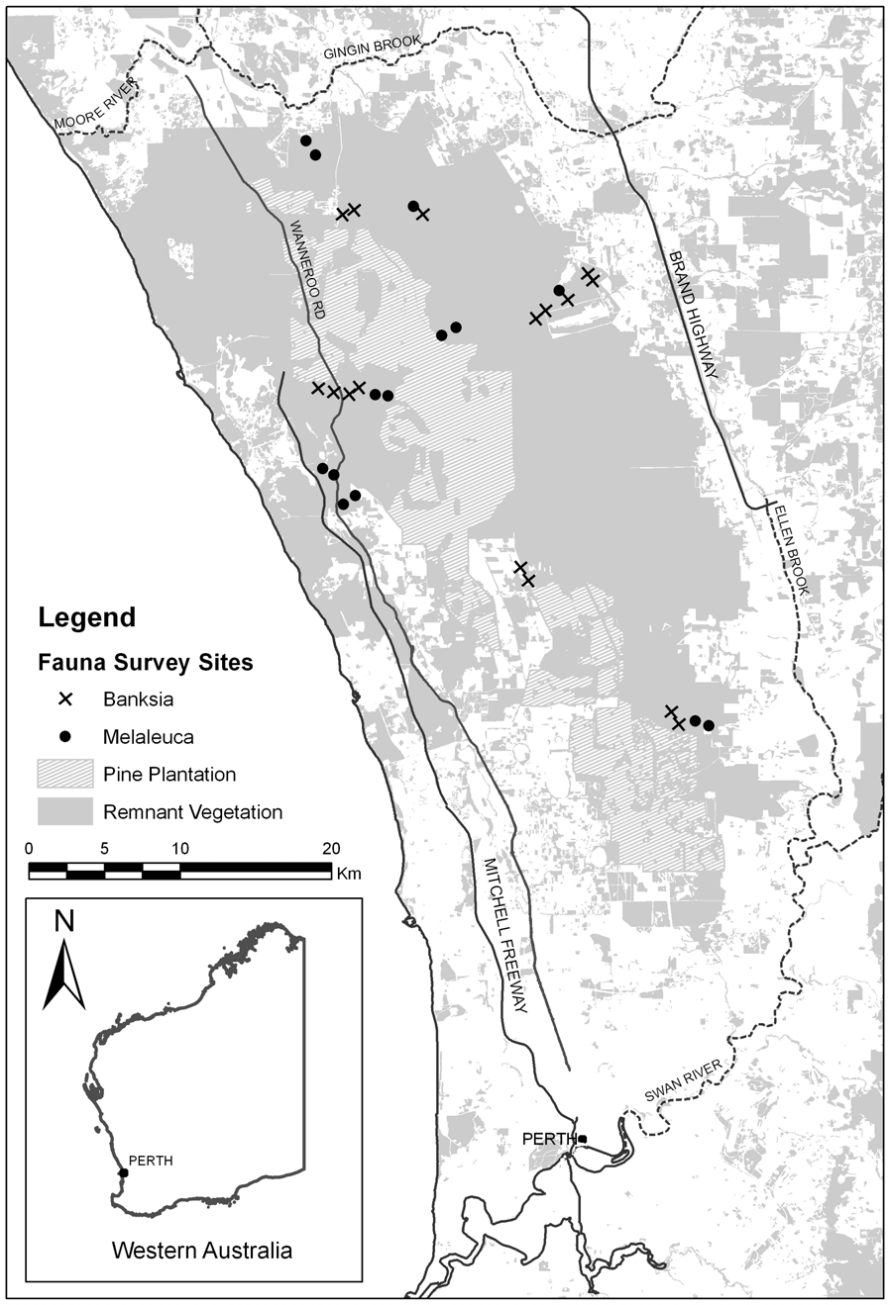
Fauna survey sites in the remnant vegetation extent surrounding Perth, Western Australia.

The study area is situated on the Swan Coastal Plain in Western Australia and extends from the Swan River in the south, to the Moore River and Gingin Brook in the north, and from the Ellen Brook in the east to the Indian Ocean in the west (Figure 1). The area is dominated by a *Banksia* overstorey with sporadic stands of *Eucalyptus* and *Allocasuarina*, and an understorey consisting mainly of low shrubs from the Myrtaceae, Fabaceae and Proteaceae families. There are many seasonal damplands, swamps and permanent wetlands, fringed by *Banksia littoralis* and *Melaleuca* trees with a variable understorey of species from the Cyperaceae, Juncaceae and Myrtaceae [37]. Although there have been large amounts of clearing for urbanisation and agriculture, the total remnant native woodland within the study area covers more than 100,000 ha. Approximately 70,000 ha are managed by the Western Australian state government (Department of Environment and Conservation, DEC) with a targeted fire-free period of 8–12 years.

The region experiences a dry Mediterranean-type climate [38], with hot dry summers (December–February) and cool wet winters (June–August), and a 100 year average of 870 mm annual rainfall recorded at the Perth meteorological station. Rainfall declines in the last 30 years have been significant, with approximately 21% less rainfall for the 1997–2003 compared to 1911–1974 [39].

### Trapping design

The sampling regime was designed to assess 30 sites in the major areas of continuous remnant bush land in the northern and eastern banksia woodland (Figure 1). Sites were selected to represent two vegetation types (banksia woodland and melaleuca damplands) and the variety of different aged habitat in terms of fire age. Vegetation associations were identified initially by spatial analysis of the Mattiske dataset [40] and field validation confirmed the dominant plant species at each site. Years since last fire (YSLF) was obtained from the 2007 Corporate Data fuel age dataset provided by Fire Management Services of the Department of Environment and Conservation. Fire events at sites (using habitat from a 1 ha polygon surrounding the pit-fall trap array) were validated using VegMachine, which incorporates Multispectral Landsat TM imagery to detect changes in reflectance which are then correlated to vegetation cover over time and analyse vegetation trends [41], [42]. Landsat estimate of vegetation cover (spectral image index) [42], [43] provided an estimate of the foliage projected cover and was calibrated with field estimates of Projected Foliage Cover (PFC). Linear trends were then calculated from the calibrated PFC images for each image date using methods described previously [41], [42]. The vegetation trend analyses have the capacity to detect changes in vegetation cover, particularly fire events which appear as abrupt increases in reflectance, and hence declines in cover.

Each site contained one pitfall trap array, with 10 pitfall traps (20 L buckets) located in a Y shape, with three pits placed along each arm radiating out from a central pit and placed at approximately seven m intervals along each arm (∼22 m). The pitfall traps were connected with 30 cm high aluminium fly wire drift fence that extended out one m beyond the last pitfall of each arm.

### Survey effort

Sites were opened for 12–20 nights in spring 2007, autumn 2008 and spring 2008, with trapping intensity varying among season and site. Logistical constraints limited the number of sites that were opened simultaneously and we ensured that sites opened at the same time were from both vegetation types and a range of time since last fire habitat to reduce sample bias within a single vegetation-fire age category. While open, all traps were checked once per day in the early morning. Captured animals were identified, measured and released onsite. Individuals were marked under the throat with a non-toxic permanent marker pen. Taxonomic nomenclature followed the Western Australian Museum. Sample-based rarefaction (species accumulation curves) were examined for the initial trapping period (spring 2007) using Estimate S [44], with horizontal curve asymptotes occurring between five to eight nights for vegetation-time since last fire groupings.

### Floristic surveys and microhabitat structure

Floristic surveys on plant species richness at each site were conducted using a 10 m×10 m quadrat established within 10 m of pit-fall trap arrays. All vascular plant species within each quadrat were recorded in spring 2008. Specimens were collected and dried in plant presses prior to identification by a botanist (D. Mickle), with nomenclature checked using the Western Australian Herbarium's plant collection database MAX. Plant species abundances were not recorded. Estimates of plant species richness at each site were determined using all plant species that were able to be differentiated as different taxa within each site.

Microhabitat variables were measured using one m×one m quadrats, placed on either side of each pit-fall trap at each site (20 quadrats per site). Attributes assessed included vegetation structure and ground substrate composition. Ground cover within the one m^2^ quadrat was estimated as a percentage of vegetation (live and dead), bareground and litter (including leaf and woody debris). Litter depth (cm) was measured using a ruler that was pressed through the litter (where relevant) until it touched a firm soil surface. To provide an index of vertical vegetation density in the understorey (within a 2 m height range), vegetation contact (both live and dead) was recorded (for height classes 0–20 cm, 20–40 cm, 40–60 cm, 60–80 cm, 80–00 cm, 100–150 cm, 150–200 cm) using a graduated pole, placed in the centre of each quadrat (referred to as touch pole counts). At the same point, canopy cover was measured using a densiometer, which calculated an approximate percentage canopy cover.

### Data Analysis

Pit-fall trapping intensity varied among sites, and we pooled the capture data from the trapping periods and performed analyses based on the relative abundance for each species and for the number of species captured from a standardised 10 trap nights. Diversity (D) and evenness (E) of the reptile assemblage at each site was calculated from these measures using Simpson's Diversity Index (1-D) [45], which ranges from 0 (low diversity) to 1 (high diversity) and Pielou's Evenness measure [46].

The years since last fire varied considerably among sites (3–36 YSLF). To examine differences based on the current prescribed burning targets, we grouped sites into two major categories based on fire age: young, those recently burnt (<12 YSLF); and old, those long unburnt (>16 YSLF). Although the categorical groupings of fire age are broad, they represent sites that are within the prescribed burning fire-free period (<12 YSLF) and sites that may be targeted for future prescribed burning, and hence reflect the different fire ages perceived by the fire management objectives. This orthogonal design included 9 replicates for the old age banksia sites, 7 replicates for the young age banksia sites, 8 replicates for the young age melaleuca sites and 6 replicates in the melaleuca sites with an old fire age.

A two-factor ANOVA (SPSS, version 17) was used to examine differences between vegetation type, fire age and any interactions for habitat variables, plant species number, reptile abundance, species richness, diversity, evenness, and the abundance of dominant species of reptiles (≥10% of observed individuals).

Community composition, defined as the average abundance (per 10 trap nights) of each species per site, was compared among factors (vegetation type and fire age) using Multi-Response Permutation Procedure (MRPP) [47], based on a rank-transformed Sorensen (Bray-Curtis) distance matrix in the statistical package, PC-ORD [48]. Rare species (species that were observed in less than three sites) were not included in the analysis. MRPP is a type of nonparametric multivariate procedure for testing differences between groups and provides an A statistic, which is the chance-corrected within group agreement, and an associated p-value [49]. Where community composition differed significantly among factors (α<0.05), non-metric multidimensional scaling (NMDS) [50] was used to graphically depict the site assemblage relationships using PC-ORD [48]. Reptile species and microhabitat and vegetation structure variables that were correlated with the NMDS axes (r^2^>0.2) are graphically depicted on the ordinations.

To examine the relationship between microhabitat variables (canopy cover, vegetation cover, bareground cover, litter cover and litter depth) and time since last fire within each vegetation type, General Additive Models (GAM) were used. We used GAMs rather than assuming linear fits, as univariate plots between the microhabitat variables and time since last fire indicated there may be non linear relationships. GAMs were fitted using the gamm function of the mgcv package in R [51], using k = 3 to avoid over-fitting and consequently unnecessary complicated models. We examined the resulting adjusted r^2^ values for these relationships.

The relative importance of the five microhabitat variables and time since last fire for predicting reptile responses (reptile abundance, species number, and the abundance of *C. adelaidensis*, *C. buchanni*, *H. quadrilineata*, *L. elegans*, *M. greyii* and *M. obscura*) was explored within each vegetation type using GAMs (as outlined above) by comparing all possible models of one, two, and three predictors. Akaike information criterion, corrected for small sample size (AICc), the associated AICc weights and adjusted r^2^ values were used to select the optimal model from this complete set [52]. These models were fitted using maximum likelihood (ML) estimation, which is appropriate for comparing models [53]. We report on the resulting models where ΔAICc<2 and adjusted r^2^ values>0.10.

### Data transformations

Habitat variables measured using percentages were adjusted by arcsine transformation of the square-root proportional data [54]. Untransformed count data (reptile abundance, species number, individual species abundance, and understorey vegetation density using touch pole counts) and litter depth were examined for normality and heteroscedasticity using box plots, Q-Q plots and residual plots. Individual species abundances, vegetation touch pole data and litter depth were square-root transformed to meet assumptions of ANOVA and Pearson's correlations.

## Results

### Patterns in Habitat Variables

Plant taxa number were higher in banksia woodland sites (Table 1; sqrt plant taxa mean (±95%CI): banksia = 7.16 (±0.40); melaleuca = 5.68 (±0.6)). A number of differences were observed in analysis of the microhabitat and vegetation structure variables between vegetation type and fire age (Table 1). Litter cover, canopy cover and litter depth were greatest in the old melaleuca sites. However, litter depth was also deeper in the old banksia sites compared to young sites (mean sqrt litter depth (±se, cm): banksia, old = 0.96 (±0.05); banksia, young = 0.69 (±0.06); melaleuca, old = 1.43 (±0.20); melaleuca, young = 0.72 (±0.06); Table 1). The vertical vegetation density in the 0–20, 20–40, 40–60, and 60–80 cm height classes was higher in the old versus young banksia sites, but lower in the old versus young melaleuca sites (e.g. mean sqrt number of touches 0–20 cm (±se): banksia, old = 1.66 (±0.12); banksia, young = 1.35 (±0.11); melaleuca, old = 0.81 (±0.12); melaleuca, young = 1.60 (±0.10); Table 1). The vertical vegetation density in the 100–150 and 150–200 cm categories were greatest in the melaleuca sites compared to banksia sites (e.g. mean sqrt number of touches 150–200 cm (±se): banksia = 0.54±(0.07); melaleuca = 1.01 (±0.13); Table 1). In addition, bareground cover was significantly greater in the young sites versus old (mean bare ground % (±se): old = 59±(0.5); young = 72 (±0.5); Table 1).

**Table 1:** ANOVA F-values for plant taxa number, microhabitat variables and vertical vegetation density showing responses to vegetation type, fire age and the interaction of vegetation type and fire age.

	Vegetation _df = 1,26_	Fire age _df = 1,26_	Vegetation * Fire age _df = 1,26_
Plant taxa number	13.774** B>M	0.003	0.009
Vegetation cover %	1.772	0.755	0.393
Litter cover %	1.013	10.920**	5.820*
Bareground %	0.747	5.627* Y>O	0.085
Canopy cover %	16.664**	8.967**	4.513*
Litter depth cm	6.665*	25.855**	4.951*
Vertical vegetation density			
0–20 cm	1.430	0.107	8.295**
20–40 cm	6.200*	4.185∧	21.178**
40–60 cm	3.252	1.510	19.513**
60–80 cm	3.510	1.510	19.513**
80–100 cm	3.141	0.356	4.005∧
100–150 cm	7.624* M>B	0.138	1.540
150–200 cm	13.824** M>B	0.042	0.142

Significant values are indicated (* *P*<0.5, ** *P*<0.01) and values approaching significance are identified (∧ 0.06>*P*≥0.05). Letters beside significant values indicate results from post-hoc Tukey HSD tests for vegetation type (B = banksia, M = melaleuca) and fire age category (O = old, >16 YSLF; Y = young, <11 YSLF).

### Abundance, Species Richness and Diversity

Pit-fall trapping at the 30 sites resulted in 1042 captures (including 35 recaptured individuals) from 33 species of lizards and snakes (Pygopidae: 7; Gekkonidae: 3; Scincidae: 15; Agamidae: 2; Elapidae: 5; Typhlopidae: 1). The most commonly caught reptiles were skinks, including the litter dwelling species *Lerista elegans* (n = 214) and *Menetia greyii* (n = 143), the tree-dwelling skink *Cryptoblepharus buchananii* (n = 106), and the small terrestrial heath dragon *Ctenophorus adelaidensis* (n = 126). The small elapid *Neelaps calonotos*, listed as a priority species requiring further monitoring under the Western Australian *Wildlife Conservation Act 1950*, was captured twice. Of the 33 reptile species observed, 12 species (4 skinks, 3 pygopids, 3 elapids, 1 gecko and 1 blind snake) were captured ≤2 times and/or were detected from ≤2 sites during surveys, and were removed from community analyses. The three elapid (*Brachyurophis semifasciata*, *Demanisa psammophis reticulata* and *Neelaps calonotus*) and one blind snake (*Ramphotyphlops australis*) species captured ≤2 times were only captured on older sites (>16 years since last fire).

**Figure 2:**
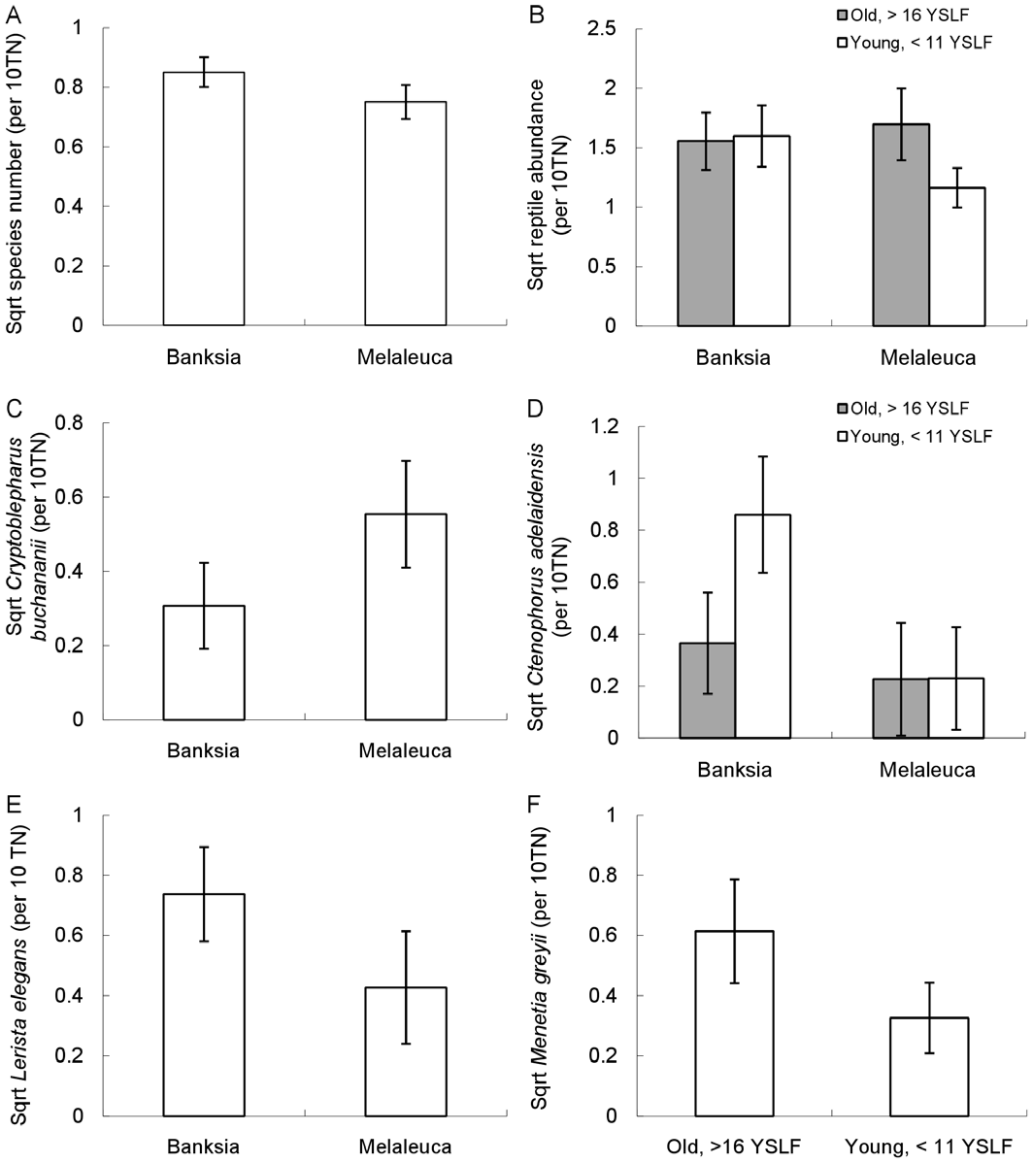
Significant differences in reptile species number and reptile abundances for habitat and fire age categories. Mean (per 10 trap nights ±95%CI) a) reptile species number b) reptile abundance and c) – e) selected individual species abundances between banksia and melaleuca habitats and f) abundance of *Menetia greyii* in old and young fire age categories.

Reptile species richness was higher in banksia woodland sites (Table 2, Figure 2a). A significant interaction term was detected for the abundance of reptiles, with fewer reptiles observed in young melaleuca sites (Table 2, Figure 2b). In contrast, the small terrestrial heath dragon *C. adelaidensis* was observed in higher abundances in young banksia sites, but was also abundant at 35 year old sites (Table 2, Figure 2d). A number of species responded to vegetation type independent of fire age. The skinks *C. buchananii*, *M. greyii* and *Hemiergis quadrilineata* were more frequently observed in melaleuca sites, while the skink *L. elegans* was more commonly observed in the banksia sites (Table 2, Figure 2c and Figure 2e; sqrt mean abundance per 10 trap nights (±95%CI): *M. greyii*, banksia = 0.38 (±0.13), melaleuca = 0.57 (±0.18); *H. quadrilineata*, banksia = 0.19 (±0.12), melaleuca = 0.40 (±0.17)). Both of the skinks *M. greyii* and *Morethia obscura* were observed in higher abundances in the older sites compared to the younger sites (Table 2, Figure 2f; sqrt mean abundance per 10 trap nights (±95%CI): *M. obscura*, old >16YSLF = 0.50 (±0.15), young <12YSLF = 0.16 (±0.09)).

**Table 2:** ANOVA F-values for reptile abundance, species richness, diversity and evenness, and individual species abundances showing responses to vegetation type, fire age and the interaction of vegetation type and fire age.

	Vegetation type _df = 1,26_	Fire age _df = 1,26_	Vegetation type * fire age _df = 1,26_
Reptile abundance	1.389	3.826	5.375*
Reptile species number	7.032* B>M	1.009	0.066
Reptile diversity	1.609	0.557	0.068
Reptile evenness	0.118	0.005	0.528
*Ctenophorus adelaidensis*	12.548**	5.239*	5.153*
*Cryptoblepharus buchananii*	7.562* M>B	0.913	1.170
*Hemiergis quadrilineata*	4.319* M>B	0.663	1.375
*Lerista elegans*	5.368* B>M	0.320	0.194
*Menetia greyii*	6.187* M>B	11.780** O>Y	2.939
*Morethia obscura*	0.332	13.040** O>Y	0.088

Significant values are in bold (* *P*<0.5, ** *P*<0.01). Letters beside significant values indicate results from post-hoc Tukey HSD tests for vegetation type (B = banksia, M = melaleuca) and fire age category (O = old, >16 YSLF; Y = young, <11 YSLF).

### Patterns in species composition

Twenty-one reptile species were detected in more than two sites and were included in community analyses. MRPP was performed on four groups based on the combination of habitat and fire age, including: melaleuca, old; melaleuca, young; banksia, old; banksia, young. MRPP detected differences in community structure between the four groups (MRPP: A = 0.201, *P*<0.001). Pair-wise comparisons indicated that the melaleuca old sites differed from the banksia old sites (*P* = 0.017), the banksia young sites (*P* = 0.001) and the melaleuca young sites (*P* = 0.047). The banksia old sites also differed to the melaleuca young sites (*P* = 0.003) and were approaching a different community structure to the banksia young sites (*P* = 0.055). Banksia young sites differed in community structure to melaleuca young sites (*P* = 0.007).

**Figure 3:**
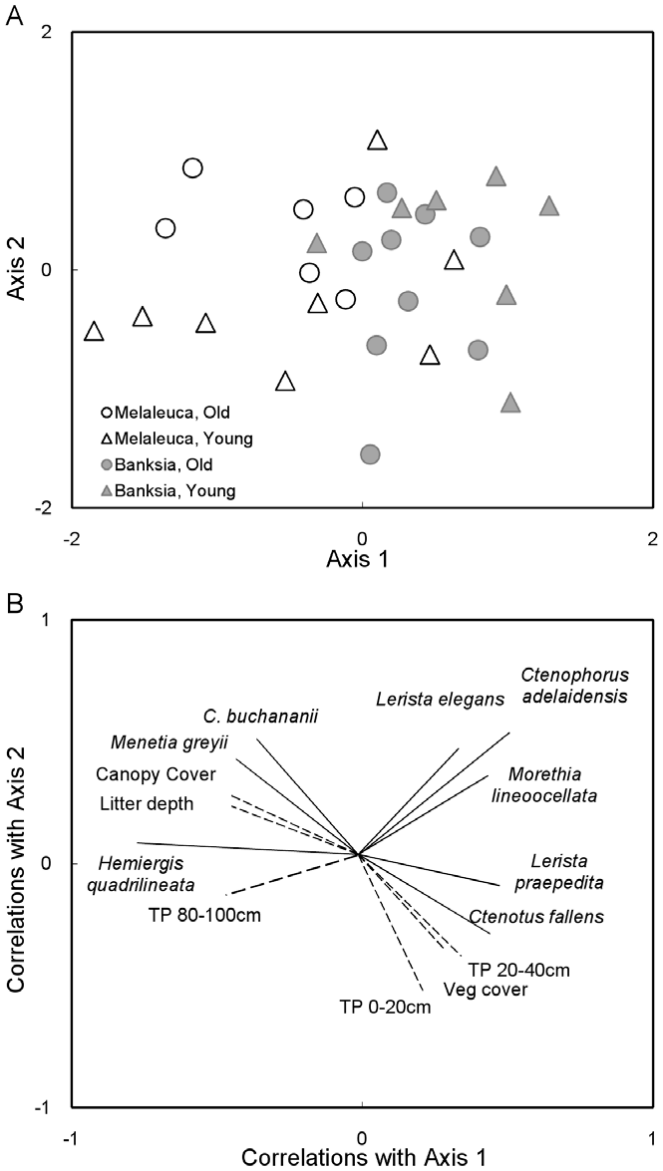
NMDS ordination (Sorensen distance measure) on the assemblage of reptiles. a) NMDS ordination of 21 reptile species at 30 sites of differing habitat (melaleuca vs banksia) and fire age (old versus young). The ordination is in two dimensions (stress = 0.193), with axis 1 and 2 cumulatively representing 75% variance (r^2^ = 0.441 and 0.310 respectively). b) Correlations of species and habitat variables (r^2^>0.2) with NMDS ordination.

NMDS ordination found a stable 2-dimensional solution representing 75% variance and a final stress value of 0.193 (Figure 3a). Vegetation types clearly contained two distinct groupings, with differences in young and old melaleuca sites also evident (Figure 3a). The separation of fire age within banksia sites was more subtle. However, banksia young sites were different to both melaleuca old and young sites (Figure 3a). One species, *H. quadrilineata* was strongly associated with melaleuca sites, regardless of fire age. Whereas the two skinks, *M. greyii* and *C. buchananii*, were associated with older melaleuca sites. No species was specifically associated with younger melaleuca sites. Species associated with banksia sites include the skinks *Ctenotus fallens*, *L. elegans*, *Lerista praepedita*, *Morethia lineoocellata*, and the dragon *C. adelaidensis*, a species that was more likely to be associated with young banksia sites. In addition, canopy cover and litter depth were associated with old melaleuca sites, while touch pole counts at 0–20 cm and 20–40 cm height categories were more likely to be associated with banksia sites (Figure 3b).

### Responses of reptiles to microhabitat variables and time since last fire

**Figure 4:**
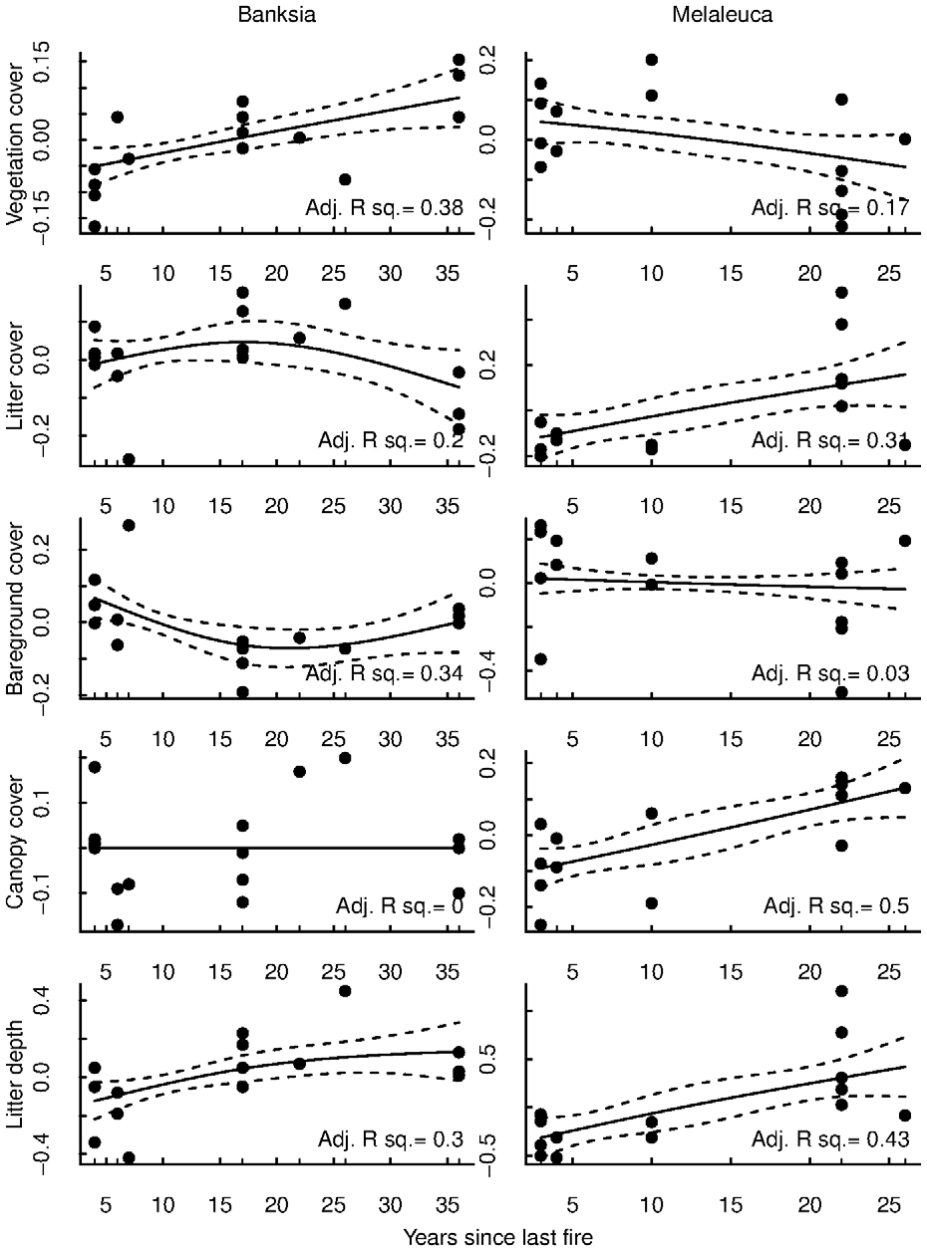
GAM relationships between time since last fire and five microhabitat variables within each vegetation type. Adjusted r^2^ values are plotted for all relationships. Values for microhabitat variables have been rescaled.

The relationship between microhabitat variables and time since last fire was dependent upon vegetation type (Figure 4). In banksia woodland sites vegetation cover increased with time since last fire (r^2^ = 0.38), but decreased in melaleuca sites (r^2^ = 0.17; Figure 4). Litter cover was highest at all sites between 15 and 25 years since last fire, but decreased in 36 year old sites in banksia woodlands (Figure 4). There were higher amounts of bareground in the young and very old banksia sites (r^2^ = 0.34), however no relationship was apparent for bareground cover in melaleuca sites (r^2^ = 0.03; Figure 4). In contrast, a strong positive relationship was observed for canopy cover in melaleuca sites (r^2^ = 0.50), whereas no relationship was detected between canopy cover and time since last fire in banksia woodlands (Figure 4). Litter depth was positively associated with time since last fire in both vegetation types (Figure 4; banksia woodlands r^2^ = 0.30; melaleuca r^2^ = 0.43).

**Figure 5:**
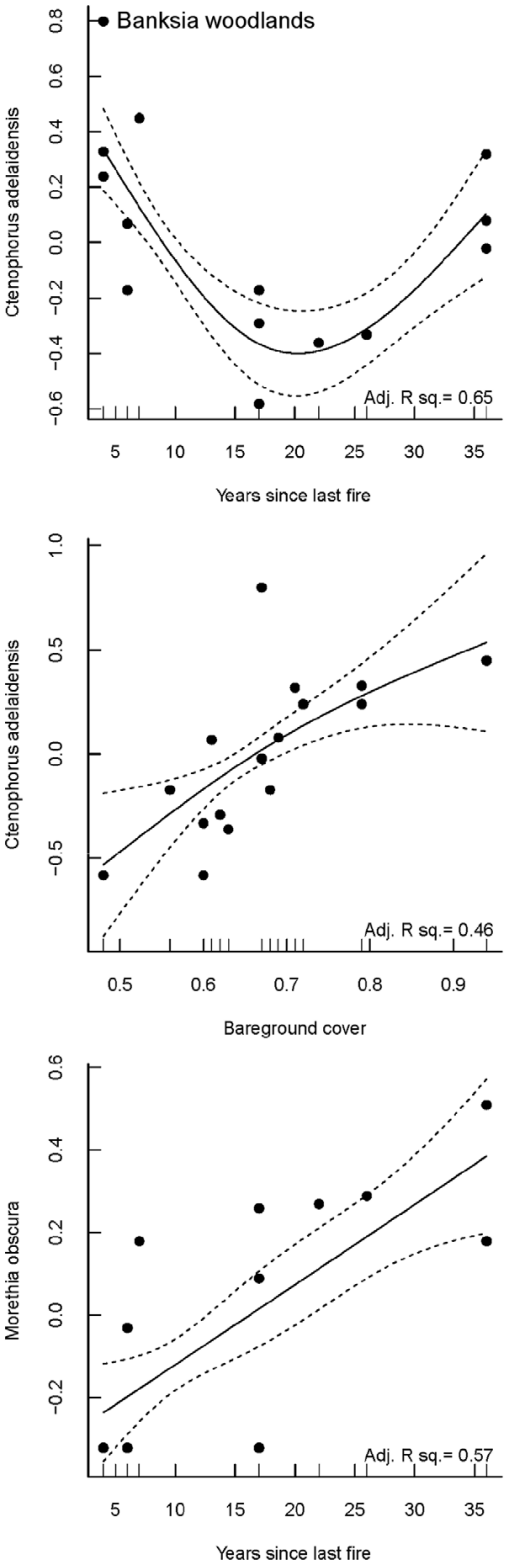
GAM relationships for the abundance of *C. adelaidensis* with time since last fire and bareground cover, and *M. obscura* with time since last fire in banksia woodlands. Adjusted r^2^ values are plotted for all relationships. Values for the abundance of reptiles are the rescaled values based on the standardised sqrt-transformed abundance per 10 trap nights.

**Figure 6:**
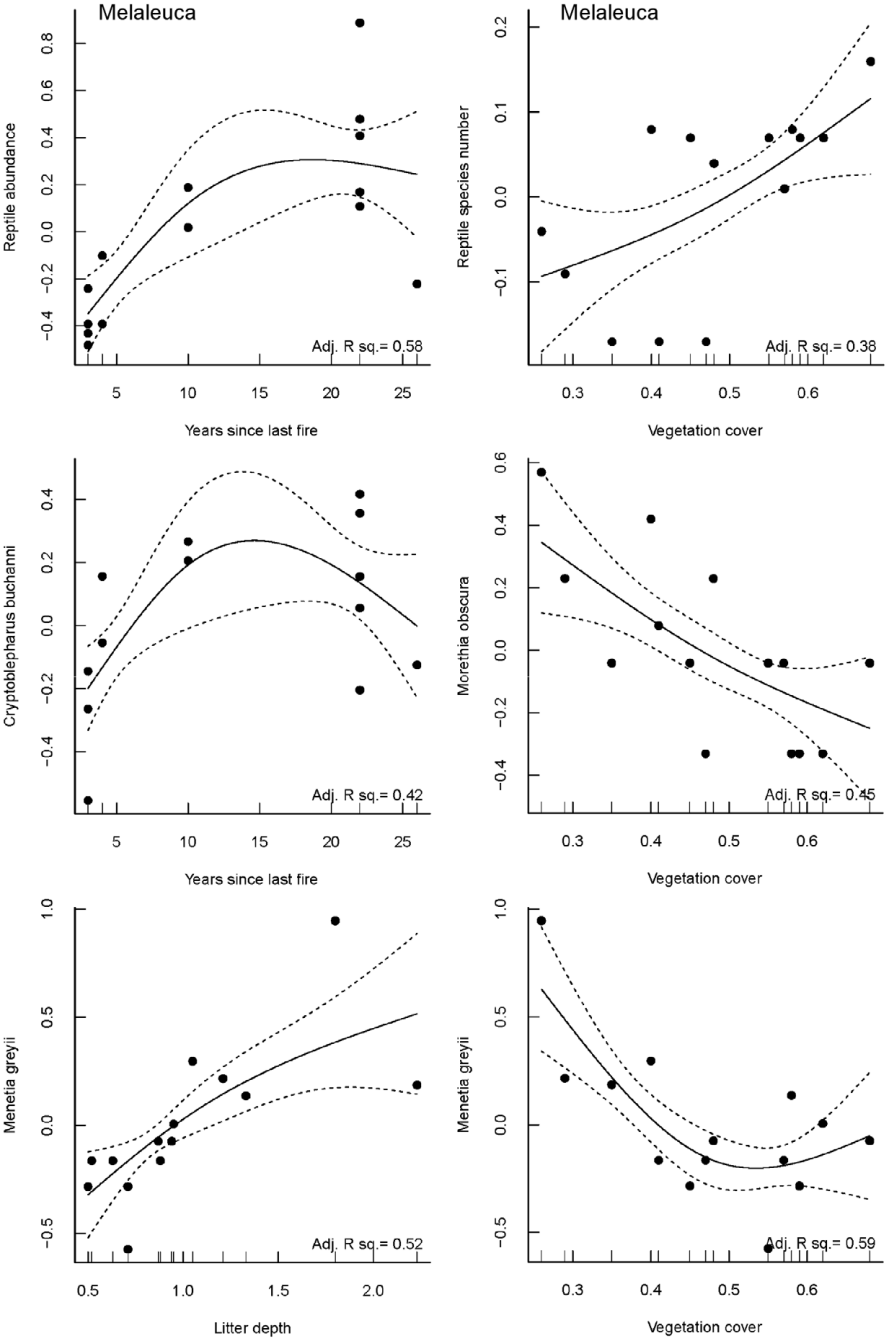
GAM relationships for reptile abundance and and the abundance of *C. buchanni* with time since last fire, reptile species number, and the abundance of *M. obscura* and *M. greyii* with vegetation cover, and *M. greyii* with sqrt-transformed litter depth. Adjusted r^2^ values are plotted for all relationships. Values for the abundance of reptiles and species number are rescaled values based on the standardised sqrt-transformed abundance/species number per 10 trap nights.

The responses of reptiles to time since last fire and microhabitat variables were dependent on the vegetation type (banksia versus melaleuca; Table 3). Top ranking models typically only included a single variable (e.g. time since last fire or canopy cover; Table 3, Figure 5 and Figure 6). For several of the reptile response variables, top ranking models had very low AICc weights and explained very little variability in the data. For example, in the banksia woodlands, six top models were observed (using ΔAICc<2) for both reptile abundance and reptile species richness, although all models carried little weight (≤0.13) and no models had an adjusted r^2^ value>0.10. In contrast, only one top model with moderate support was detected for reptile abundance (AICc weight = 0.39; adjusted r^2^>0.58) and species number (AICc = 0.37; adjusted r^2^ = 0.38) with time since last fire and vegetation cover respectively in the melaleuca damplands (Table 3). In this vegetation type, reptile abundance showed a strong curvilinear relationship with time since last fire, with reptile abundance lowest in the very young and very old fire ages (Figure 6). Reptile species richness in the melaleuca sites increased with the amount of vegetation cover (Figure 6).

**Table 3:** Top-ranking generalised additive models (GAM) for reptile response variables with time since last fire (YSLF) and microhabitat variables within each vegetation type.

Variable	YSLF	Veg %	Litter %	Bare %	Canopy %	Litter depth	ΔAICc	AICc weights	Adjusted R^2^
Banksia woodlands									
Reptile abundance							-	-	-
Reptile species number							-	-	-
*Ctenophorus adelaidensis*	X						0.00	0.40	0.65
				X			1.88	0.16	0.46
*Cryptoblepharus buchanni*					X		0.00	0.22	0.21
*Hemiergis quadrilineata*			X				0.00	0.15	0.11
*Lerista elegans*					X		0.00	0.15	0.11
*Menetia greyii*	X						0.00	0.15	0.11
					X		0.08	0.14	0.11
			X		X		1.93	0.06	0.33
*Morethia obscura*	X						0.00	0.48	0.57
Melaleuca damplands									
Reptile abundance	X						0.00	0.39	0.58
Reptile species number		X					0.00	0.37	0.38
*Ctenophorus adelaidensis*							-	-	-
*Cryptoblepharus buchanni*			X				0.00	0.19	0.30
						X	0.34	0.16	0.27
	X						0.70	0.14	0.42
					X		1.43	0.09	0.19
				X			1.74	0.08	0.18
*Hemiergis quadrilineata*			X				0.00	0.19	0.23
						X	0.09	0.18	0.23
*Lerista elegans*							-	-	-
*Menetia greyii*						X	0.00	0.17	0.52
		X					0.05	0.17	0.59
			X				0.30	0.15	0.49
		X				X	1.52	0.08	0.69
*Morethia obscura*		X					0.00	0.44	0.45

Only the models with a ΔAICc<2 and an adjusted r^2^>0.10 are shown. Variables detected for each model are indicated with an ‘X’. Microhabitat variables include: YSLF = years since last fire, Veg = vegetation cover and Bare = bareground cover.

The top model for both the skink *M. obscura* and the dragon *C. adelaidensis* in banksia woodlands was the variable times since last fire (Table 3; Figure 5). The abundance of *M. obscura* was positively associated with time since last fire in banksia woodlands (adjusted r^2^ = 0.57; Figure 5). Whereas, the abundance of *C. adelaidensis* showed a strong (adjusted r^2^ = 0.65) curvilinear response with time since last fire (Table 3; Figure 5), with highest abundance in the very young (4–6 YSLF) and very old sites (36 YSLF). In addition, bareground cover (adjusted r^2^ = 0.46) was also included in the top models for *C. adelaidensis* in banksia woodlands, indicating that both of these variables are potential predictors of the abundance of this small dragon (Figure 5). However, in melaleuca sites, all models had low AICc weights (<0.15) and explained very little variability (r^2^<0.05). In melaleuca sites, the abundance of *M. obscura* was best explained by vegetation cover (adjusted r^2^ = 0.45), whereby an increase in vegetation cover, was associated with a decrease in the abundance of this skink (Figure 6).

A positive relationship between canopy cover and the abundance of the skink *C. buchanni* was detected as the top model in banksia woodland (adjusted r^2^ = 0.21; Table 3). All variables, with the exception of vegetation cover, were included in the top models for this species in melaleuca damplands, although time since last fire explained the most variability (adjusted r^2^ = 0.42; Table 3; Figure 6), with a curvilinear response with lowest abundances of this skink observed in very young (3 YSLF) and very old sites (26 YSLF).

For the skinks *H. quadrilineata*, and *M. greyii* in banksia woodlands, and for *L. elegans* in both banksia woodlands and melaleuca damplands, six top models with very low weights were also observed, indicating that time since last fire and the microhabitat variables measured all have limited ability to explain variability in abundance of these species. Litter cover (adjusted r^2^ = 0.23) and litter depth (adjusted r^2^ = 0.23) were identified in top models for *H. quadrilineata* in melaleuca damplands (Table 3). In melaleuca damplands, the abundance of *M. greyii* was best explained by litter depth (adjusted r^2^ = 0.52), vegetation cover (adjusted r^2^ = 0.59), litter cover (adjusted r^2^ = 0.49) and a combination of both litter depth and vegetation cover (adjusted r^2^ = 0.69) (Table 3; Figure 6).

## Discussion

### Reptile communities and fire management targets

The responses of microhabitat structure and reptiles to fire age are strongly driven by vegetation types. The differences in microhabitat structure among fire ages were particularly pronounced in melaleuca sites. For example, litter depth was greater in the older sites, but the differences between old and young melaleuca sites was more than double the differences between old and young banksia sites. Similarly, the differences in vertical vegetation density were more pronounced in melaleuca sites. The older aged banksia sites contained higher vegetation density (<80 cm), whereas the younger melaleuca sites contained higher vegetation density (<80 cm). Unsurprisingly, the banksia woodland sites were more floristically diverse compared to the melaleuca damplands. Much of the banksia woodland diversity is contained in the highly variable understorey <60 cm [55], suggesting that vegetation structure in young melaleuca sites are only from a few species recovering post-fire, potentially limiting the diversity of structure.

Reptile communities also varied between vegetation types and fire ages. Young melaleuca habitat tended to contain fewer reptiles, and had few species associated with them. Although some differences were also detected among young and old banksia sites, these were less pronounced. However, our results indicate that three of the six most frequently detected reptile species (*C. adelaidensis*, *M. greyii*, and *M. obscura*) responded to fire age, and two additional skinks, *C. fallens* and *C. buchananii*, were associated with older fire ages within a particular habitat type. Responses of reptiles to fire age may be associated with microhabitat attributes that also vary between fire age categories [17], [21].

These results provide relevant empirical data on how biodiversity may be affected by fire ages determined by prescribed burning management targets. The relationship of fauna with fire varies depending on the vegetation type, and fire regimes should ideally be applied to the specific vegetation type at a local scale. On the Swan Coastal Plain, recently burnt melaleuca sites are fairly species-depauperate, indicating that the reptile communities of melaleuca sites are susceptible to the impacts of fire. We recommend active protection of the older-aged melaleuca sites from fire. Although, the logistical constraints of applying fire management in this manner are likely to be challenging.

The current prescribed burning regime, aiming for a fire-free period of 8–12 years, targets older-aged sites, potentially removing critical habitat for some species, especially the older-aged melaleuca sites. At a landscape level, the remnant vegetation in the GSS currently has a mosaic of fuel ages, which is highly skewed towards the more recently burnt (∼60% of the area is ≤6 years since last fire) [56]. In addition, the spatial distribution of post-fire age for the 70,000 ha of conservation estate is also highly clustered, with large areas of similar post-fire ages grouped together [56]. One of the greatest challenges facing land managers in this area, as with other regions where the remnant vegetation adjoins urban areas, is juggling the necessity of reducing fire risk while maintaining conservation values [4].

### Seral responses of reptiles to fire and microhabitat variables

Our study has highlighted how time since last fire and microhabitat variables may influence species' abundances. Of the eight reptile response variables examined, time since last fire was identified as an important predictor for four variables (reptile abundance, *C. adelaidensis*, *C. buchanni* and *M. obscura*) in one of the vegetation types. In addition, various microhabitat variables were identified in top models for at least one vegetation type for reptile species number, *C. buchanni*, *M. greyii*, *M. obscura* and *H. quadrilineata*. In both vegetation types, time since last fire and microhabitat variables were unable to explain much variability in the abundance of *L. elegans* (highest r^2^ = 0.11).

The responses of microhabitat variables to time since last fire also varied between vegetation types. For example, vegetation cover positively increased with time since last fire in banksia woodlands, but decreased in melaleuca damplands. Such opposite responses are likely a function of plant species composition, with vegetation cover in young melaleuca sites dominated by few species. As time since last fire increases, the understorey vegetation cover may decline in response to an increase in canopy cover. Only a few of the relationships among microhabitat variables and time since last fire were very strong (highest r^2^ = 0.50), indicating the composition of microhabitat in these vegetation types are likely to driven by additional factors.

Changes in the abundance of reptiles following burning is often linked to fire-induced changes in the resource availability of the postfire environment [20], [21], [57]. Some reptile species may prefer the early post-fire habitat, particularly those that prefer bareground [20], [58], [59], [60]. In our study, the terrestrial dragon, *C. adelaidensis*, was more abundant in the young and very old banksia sites and sites with high amounts of bareground. Litter accumulation in banksia woodlands (which shows an inverse relationship with bareground) increases until approximately 20 years since last fire, after which the litter cover and depth declines and plateaus respectively. Rates of ground litter fuel accumulation are different for different components of fuel [61]. Ground litter fuel in banksia woodlands accumulates greatest within the first 4–6 years following a fire and remains stable for 6–20 years post fire [62]. Our research concurs with this finding, and further suggests that litter accumulation rates in sites greater than 25 years since last fire decline, and the amounts of bareground increase. The resurgence in abundance of *C. adelaidensis* at older sites was possibly in response to this change in litter accumulation. A similar response to combinations of time since fire and microhabitat changes has been observed for the rodent *Pseudomys novaehollandiae* [63].

As reptile abundance was lowest in very young (<5 YSLF) melaleuca sites, burning may have modified elements of the microhabitat in a manner undesirable to some species. The young melaleuca sites had reduced litter depth, litter cover and canopy cover, and a limited number of plant species. Typically, litter-associated lizards, such as *M. greyii* in our study, respond strongly to the removal of litter and are usually observed in high abundance in the least-disturbed sites, with their density often correlated with microhabitat variables, such as litter cover [21], [25]. However, time since last fire was the single best predictor of reptile abundance in melaleuca sites, indicating that microhabitat changes alone are unlikely to be driving this relationship, at least for the variables we measured. Because reptiles tend to occupy sites with suitable thermal, shelter, and food resources [17], [21], [57], it is likely that burning has altered some form of ecological interaction, either by changing prey availability, predation susceptibility or thermal requirements.

Similarly, the abundance of *M. obscura* in banksia woodlands is best described by time since last fire, rather than microhabitat variables, with the highest numbers of this skink detected in very old sites. In contrast, in jarrah forests in south-west Western Australia, this species was only recorded in restored mine sites that had been thinned and recently burnt [64]. The abundance of *M.obscura* may be driven by broader scale ecological process (e.g. thermal requirements) that fire affects in various ways depending on the ecosystem. Knowledge of the ecology and natural history of small lizards is depauperate and hampers our interpretation of species' responses to disturbances.

For prescribed burning to be effective for conservation purposes, land managers require understanding of the impacts of fire on biota, and clear management targets regarding burning practices and ecological objectives [4]. This study has contributed towards a greater understanding of the responses of reptiles to fire regimes in south-west Western Australia. Specifically, we recommend the active protection of older-aged melaleuca sites that are interspersed within the banksia woodland matrix. In addition, based on the occurrences of some rarely captured species (including *Neelaps calonotus*) we recommend retaining older-aged banksia sites (>12 years since last fire) that are currently being targeting for prescribed burning. Our work will contribute to the development of ecological burning regimes incorporating both flora and fauna responses to fire. Recommendation for burning rotations that will retain biodiversity values, reduce wildfire risk and be spatially variable can be developed by conservation and fire managers.
